# Antibiotic susceptibility and architecture of *Staphylococcis aureus* and *Staphylococcus epidermidis* biofilms

**DOI:** 10.1186/1753-6561-5-S6-P199

**Published:** 2011-06-29

**Authors:** DC Coraca-Huber, M Fille, J Hausdorfer, K Pfaller, M Nogler

**Affiliations:** 1 Experimental Orthopedics, Medical University Innsbruck, Austria; 2 Hygiene and Medical Microbiology, Medical University Innsbruck, Innsbruck, Austria; 3 Histology And Embryology, Medical University Innsbruck, Innsbruck, Austria

## Introduction / objectives

The infection associated to metal surfaces or dead tissues like bone grafts, can be fatal for the patient and presents a major financial burden for the economy. The adhering bacteria in these cases can evade host defences by forming biofilms. For this reason, the prevention of bacterial colonization and control of implant associated infections are of special interest.

## Objectives

Growth of *S.aureus* and *S. epidermidis* biofilms *in vitro* for antibiotic susceptibility tests and investigation of architecture.

## Methods

*S. aureus* and *S. epidermidis* biofilms were grown over MBEC^®^ (modified microtiter plates). Antibiotic susceptibility tests were carried out using gentamicin, vancomycin, rifampicin, fosfomycin, clindamycin and linezolid. Cell counting, opacity density (OD_620_) and scanning electronic microscopic (SEM) analysis were carried out.

## Results

The counting of viable cells after antibiotic exposition and OD_620_ showed significant efficacy of rifampicin and gentamicin against *S. epidermidis* biofilms and rifampicin against *S. aureus* biofilms compared to other antibiotics. SEM images showed proteic material in contact with cells which can be related to the proteic membrane characteristic of the biofilms structure.

## Conclusion

The method for the development of bacterial biofilm *in vitro* using MBEC^®^ plates is efficient and relatively fast. Gentamicin and rifampicin are good candidates for control of implant associated infections.

## Disclosure of interest

None declared.

